# Visualizing the strong field–induced molecular breakup of C_60_ via x-ray diffraction

**DOI:** 10.1126/sciadv.adz1900

**Published:** 2025-11-21

**Authors:** Kirsten Schnorr, Sven Augustin, Ulf Saalmann, Georg Schmid, Arnaud Rouzée, Razib Obaid, Andre AlHaddad, Nora Berrah, Cosmin I. Blaga, Christoph Bostedt, Manuel Cardosa-Gutierrez, Gabriella Carini, Ryan Coffee, Louis F. DiMauro, Philip Hart, Yuta Ito, Katharina Kubicek, Yoshiaki Kumagai, Jochen Küpper, Yu Hang Lai, Hannes Lindenblatt, Ruth A. Livingstone, Severin Meister, Robert Moshammer, Koji Motomura, Thomas Möller, Kaz Nakahara, Timur Osipov, Gaurav Pandey, Dipanwita Ray, Francoise Remacle, Daniel Rolles, Jan Michael Rost, Ilme Schlichting, Rüdiger Schmidt, Simone Techert, Florian Trost, Kiyoshi Ueda, Joachim Ullrich, Marc J.J. Vrakking, Julian Zimmermann, Claus Peter Schulz, Thomas Pfeifer

**Affiliations:** ^1^ Paul Scherrer Institut, 5232 Villigen, Switzerland.; ^2^ Max-Planck-Institut für Kernphysik, 69117 Heidelberg, Germany.; ^3^ J.R. Macdonald Laboratory, Department of Physics, Kansas State University, Manhattan, KS 66506, USA.; ^4^ Max-Planck-Institut für Physik komplexer Systeme, 01187 Dresden, Germany.; ^5^ Max Born Institut, 12489 Berlin, Germany.; ^6^ University of Connecticut, Storrs, CT 06269, USA.; ^7^ Argonne National Laboratory, Argonne, IL 60439, USA.; ^8^ The Ohio State University, Columbus, OH 43210, USA.; ^9^ École Polytechnique Fédérale de Lausanne, 1015 Lausanne, Switzerland.; ^10^ Theoretical Physical Chemistry, RU Molsys, University of Liege, 4000 Liege, Belgium.; ^11^ SLAC National Accelerator Laboratory, Menlo Park, CA 94025, USA.; ^12^ Brookhaven National Laboratory, Upton, NY 11973, USA.; ^13^ IMRAM, Tohoku University, 980-8577 Sendai, Japan.; ^14^ Deutsches Elektronen-Synchrotron DESY, 22607 Hamburg, Germany.; ^15^ Centre for Ultrafast Imaging, Universität Hamburg, 22761 Hamburg, Germany.; ^16^ Max-Planck-Institut für biophysikalische Chemie, 37077 Göttingen, Germany.; ^17^ Department of Physics, Universität Hamburg, 22761 Hamburg, Germany.; ^18^ Center for Free-Electron Laser Science, Deutsches Elektronen-Synchrotron, 22607 Hamburg, Germany.; ^19^ Technische Universität Berlin, 10623 Berlin, Germany.; ^20^ Max-Planck-Institut für Medizinische Forschung, 69120 Heidelberg, Germany.; ^21^ Technische Universität Dresden, 01062 Dresden, Germany.; ^22^ Physikalisch Technische Bundesanstalt, 38116 Braunschweig, Germany.; ^23^ Eidgenössische Technische Hochschule Zürich, 8092 Zürich, Switzerland.

## Abstract

Laser-driven dynamics in polyatomic molecules poses a complex many-body problem. Understanding intense light-matter interaction is crucial for steering intramolecular quantum dynamical processes. Here, we record time-resolved x-ray diffraction images of $C_{60}$ molecules during and after their interaction with intense near-infrared fields, giving direct access to structural changes of the molecules and their fragmentation in real time. Tuning the intensity of the excitation pulses, we uncover a transition from a weak-field regime of excited but stable molecules to a high-field regime dominated by Coulomb explosion. In the transition region, the molecules expand by up to 50% of their initial size within just 140 fs, with major fragmentation only setting in afterward. This work demonstrates that x-ray diffractive imaging is capable of retrieving time-resolved structural information of large molecules reshaped by intense laser fields. Laser-driven fragmentation is a first step toward observing molecular processes modified by laser fields of increasing intensity.

## Teaser

X-ray laser illuminates how intensity-tunable light reshapes $C_{60}$ molecules and their fragmentation dynamics.

## INTRODUCTION

Lasers can steer chemical reactions through the manipulation of molecular potential-energy surfaces. This control exploits the dynamic Stark effect of molecules in strong optical laser fields (*1*, *2*). Light-induced potential-energy curves have first been observed in the 1990s in $H_{2}^{+}$ manifesting as softening (*3*) or hardening (*4*) of a molecular bond. Even conical intersections can be induced by light, allowing to control the outcome of chemical reactions, such as photodissociation (*5*) and photoisomerization (*6*). The larger the molecular system to be steered, the more important are direct imaging methods to observe the effect of such laser control.

Fullerenes (*7*, *8*) are prototype systems to study fundamental light-matter interaction processes such as energy dissipation by efficient coupling of many electronic and nuclear degrees of freedom spanning timescales from atto- to microseconds (*9*). The response of gaseous $C_{60}$ molecules to intense femtosecond (fs) laser pulses keeps attracting substantial interest both experimentally (*10*–*12*) and theoretically (*13*, *14*). The ionization and fragmentation patterns of $C_{60}$ following irradiation with intense laser fields show the simultaneous creation of stable highly charged $C_{60}^{n+}$ ions (*15*) along with the fragmentation into, e.g., $C^{+},\ldots ,C_{11}^{+}$ ions (*10*, *16*). The breakup of the carbon cage into smaller and smaller fragments is proposed to proceed via a complex chain of bond ruptures and formations, and other nuclear rearrangement reactions. Often, the interpretation of results involved dominant excitation of either the symmetric breathing $a_{g}\left(1\right)$ (*14*, *17*–*19*) or the oblate-prolate $h_{g}\left(1\right)$ (*15*, *20*, *21*) mode, with vibrational periods of 67 and 122 fs, respectively.

The experimental results have up to now been almost exclusively based on charged-particle spectroscopy techniques, detecting electrons and ions nano- to microseconds after the laser interaction. Thus, the formation of transient structures and reactions taking place on the way to the detector, such as electron-ion recombination, remain hidden, and information about neutral fragments is lost. Femtosecond time-resolved x-ray coherent imaging is able to overcome these limitations and has recently become feasible on dilute samples with the advent of free-electron lasers (FELs), which provide sufficiently short and intense x-ray pulses with ~10^12^ photons per pulse (*22*–*26*). Here, we use elastic x-ray scattering to image the response of a gas-phase $C_{60}$ ensemble undergoing near-infrared (NIR) laser–induced deformations as a function of time, directly observing the initial expansion and subsequent fragmentation of the molecules.

## RESULTS

**Fig. 1.**
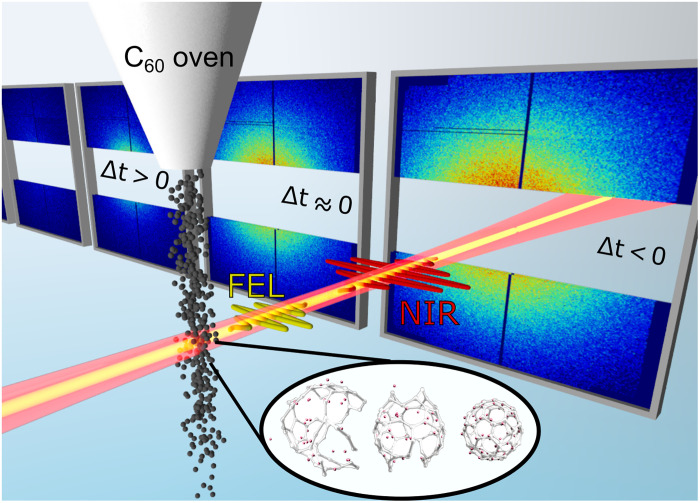
Experimental scheme. An ensemble of $C_{60}$ molecules is excited and ionized by an NIR pulse and probed by an FEL pulse via elastic x-ray scattering. Experimental scattering patterns at different delays Δt are shown for the intermediate NIR intensity. The inset contains three examples of computed structures resulting from the exposure to the NIR pulse.

The experimental scheme we used in order to investigate the NIR-induced dynamics of $C_{60}$ molecules is illustrated in Fig. 1. An NIR fs laser pulse with central wavelength of 800 nm ionizes the $C_{60}$ molecules to a degree that can be adjusted by varying the NIR laser intensity. The NIR intensity was calibrated in situ using ion time-of-flight (TOF) spectra of ionized and fragmented $C_{60}$ molecules. We monitor the structural rearrangement of the ionized molecules by time-resolved x-ray coherent imaging using an intense fs x-ray FEL pulse at a photon energy of 1.8 keV. Scattering images for each time delay have to be averaged over thousands of shots due to the low scattering cross section of gas-phase $C_{60}$ molecules. More details on the experimental parameters can be found in the “Experiment” section under Materials and Methods, as well as the Supplementary Materials.

Since $C_{60}$ molecules are spherical, we need a model that allows us to describe scattering off spherical objects to interpret our recorded scattering patterns. For this purpose, we perform Guinier fits to the averaged scattering patterns, a method that is routinely used in x-ray scattering to determine the size of molecules. We followed the description by Guinier and Fournet given in section 2.1.2.6 of (*27*), where scattering off different geometrical objects is derived. More details can be found in the “Guinier fit” section in the Supplementary Materials. The scattering intensity I(q,R) for a spherical object is given by

$$I\left(q,R\right)=I_{e}\left(q\right)F^{2}\left(q,R\right)$$ (1)

$$\begin{array}{c} F\left(q,R\right)\overset{!}{=}F_{\text{Guinier}}\left(q,R\right)\\ =\underset{N}{\underbrace{V_{\text{sph}}\left(R\right)\rho_{e}\left(R\right)}}\left[3\frac{\text{sin}\left(qR\right)-qR\text{cos}\left(qR\right)}{{\left(qR\right)}^{3}}\right] \end{array}$$ (2)

Here, q is the momentum transfer, R the radius of the scattering object, $V_{\text{sph}}\left(R\right)$ the volume of the target sphere and $\rho_{e}\left(R\right)=\frac{N}{V_{\text{sph}}\left(R\right)}$ the average electronic scatterer density of N scatterers. We can rewrite Eq. 1 introducing the Guinier amplitude A

$$I\left(q,R\right)=A{\left[3\frac{\text{sin}\left(qR\right)-qR\text{cos}\left(qR\right)}{{\left(qR\right)}^{3}}\right]}^{2}$$ (3)

$$A=I_{e}\left(q\right)N^{2}$$ (4)

which has a quadratic dependence to the number of scatterers N.

The spherical symmetry of $C_{60}$ molecules in a strong laser field may be deformed to ellipsoidal with the symmetry axis being the laser-polarization direction. To account for deformation imposed by the laser pulse, we have used the Guinier fit for ellipsoidal objects in our analysis, where R becomes dependent of $R_{\text{pol}}$ and $R_{\text{equ}}$ , the two radii of an ellipse (cf. the “Guinier fit” section in the Supplementary Materials).

**Fig. 2.**
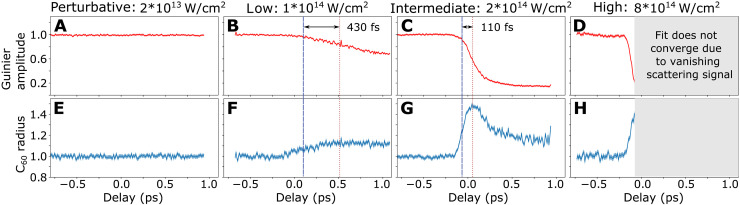
Measured x-ray scattering signals as a function of NIR pump–x-ray probe delay. The normalized Guinier amplitude for the perturbative, low, intermediate and high NIR intensity (**A** to **D**) and the corresponding Guinier radii in units of the ground state $C_{60}$ radius are plotted (**E** to **H**). Error bands indicate 1σ uncertainty (cf. the Supplementary Materials section “Uncertainty estimation”). Dashed blue lines mark the delays when the Guinier radii have increased to half of their maximal expansion and red dotted lines indicate the delays when the Guinier amplitudes have dropped to half of their respective minima.

We perform Guinier fits to the ensemble-averaged diffraction patterns for each 10 fs time bin. In particular for large NIR intensities, molecules fragment and the shape of each individual molecule will no longer be spherical or ellipsoidal for positive delays (cf. Fig. 1). However, because of the averaging over multiple thousands shots and thus many orientations of the molecular breakup pattern, we assume that the ensemble-averaged fragmentation pattern adds up to being spherical or ellipsoidal. The fitted Guinier amplitude A (Fig. 2, A to D) is proportional to the amount of scattered photons per solid angle and thus gives a measure of the degree of fragmentation and ionization of the molecules. A breakup of an intact $C_{60}$ molecule into multiple moieties reduces the number of scatterers $N_{i}$ per moiety and in turn the Guinier amplitude A , which is proportional to $N_{i}^{2}$ . The Guinier amplitude was normalized to that of intact molecules obtained for negative delays. The fitted Guinier radius R is proportional to the radius of the $C_{60}$ molecules and was normalized to the ground-state radius, also determined from data taken for negative delays (Fig. 2, E to H). Figure 2 shows the delay-dependent Guinier amplitudes and radii for four different NIR intensities [referred to as perturbative ( $2\times 10^{13}\frac{W}{{\text{cm}}^{2}}$ ), low ( $1\times 10^{14}\frac{W}{{\text{cm}}^{2}}$ ), intermediate ( $2\times 10^{14}\frac{W}{{\text{cm}}^{2}}$ ), and high ( $8\times 10^{14}\frac{W}{{\text{cm}}^{2}}$)].

**Fig. 3.**
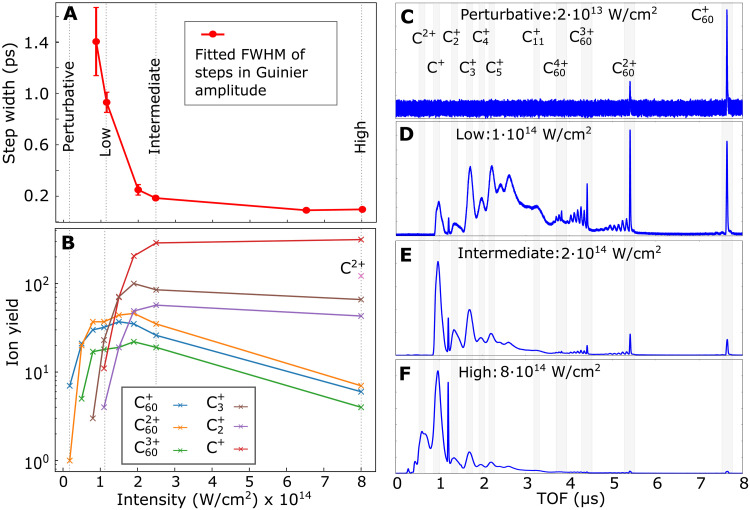
NIR intensity-dependent dynamics. (**A**) FWHM of an error-function fit to the delay-dependent Guinier amplitude for different NIR intensities. (**B**) Charge-state yields from TOF spectra of selected ion species as a function of the NIR intensity. (**C** to **F**) TOF spectra recorded with only the NIR laser at the (C) perturbative, (D) low, (E) intermediate, and (F) high NIR intensity. Characteristic fragments are labeled on top of the respective peaks. The progression of peaks toward the small TOF side of the $C_{60}$ cations corresponds to cations which have lost a single or multiple $C_{2}$ fragments (*11*). The sharp peak between $C^{+}$ and $C_{2}^{+}$ corresponds to $H_{2}O^{+}$ from ionization of residual gas.

At the perturbative NIR intensity, no dynamical response is observed in the experimental x-ray scattering patterns (Fig. 2, A and E). The corresponding TOF spectrum in Fig. 3C contains mainly $C_{60}^{+}$ cations, but no charged fragments. While the TOF spectra can only give information about ions detected microseconds after the interaction has taken place, x-ray coherent imaging is sensitive to in situ structural changes of both neutral and charged particles. Thus, the absence of delay-dependent features in Fig. 2 (A and E) indicates that the molecular structure is not altered on a picosecond timescale irrespective of whether the molecules were ionized or remained neutral during the NIR interaction. At the low intensity, the measurements of the Guinier amplitude as a function the delay, shown in Fig. 2B, provide a remarkably different view on the NIR-induced $C_{60}$ dynamics than does the NIR-induced ion TOF spectrum shown in Fig. 3D. The TOF spectrum at the low intensity (Fig. 3D) might give the impression that fragmentation is already the dominant relaxation pathway given the amount of charged fragments. In contrast, the small drop of <30% in the Guinier amplitude (Fig. 2B) shows that the majority of molecules remains intact within the observed delay range. This apparent discrepancy is explained by the fact that the TOF measurement is substantially more sensitive to small charged fragments compared to fullerene species (*28*) and blind to neutral molecules and fragments.

From NIR intensities $1\times 10^{14}\frac{W}{{\text{cm}}^{2}}$ , the Guinier amplitude drops for positive NIR pump–x-ray probe delays by an amount and at a rate that increases with the NIR intensity (Fig. 2, B to D). This is due to ionization-induced disintegration of a growing number of molecules. Between the low and intermediate intensity, the amplitude drop is particularly pronounced (Fig. 3A) and levels out afterward. The strong contrast from predominantly stable molecules at the low intensity to mostly fragmented molecules at the intermediate intensity shows a behavior similar to ionization ignition in strong field–ionized atomic clusters (*29*). There, ionization and consequently fragmentation is strongly enhanced when the laser intensity reaches the single ionization threshold: A burst of electrons, driven by the laser field, recollides multiple times and drives avalanche ionization via electron impact (*30*). The TOF spectra show a corresponding transition from a majority of stable $C_{60}^{n+}$ and larger fragment ions at the low intensity to predominantly small ionic fragments ( $C_{2}^{+}$ , $C^{+}$ , etc.) at the intermediate intensity (Fig. 3B). Both the intensity-dependent Guinier amplitude step widths (Fig. 3A) and the small fragments yield (Fig. 3B) show their steepest descent and ascent, respectively, in the region between low and intermediate intensity supporting the ionization-ignition analogy. In addition, Coulomb explosion driven by cascade hopping of electrons could contribute to the fast amplitude drop (*31*).

**Fig. 4.**
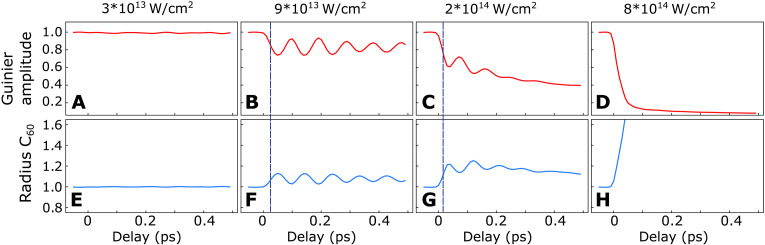
Calculated delay-dependent scattering signals, masked with the same detector geometry and analyzed like the experimental data. The Guinier amplitude for the four different NIR intensities (**A** to **D**) are shown along with the corresponding Guinier radii in units of the ground state $C_{60}$ radius (**E** to **H**). Dashed blue lines mark the delays when the Guinier radii have increased to half of their first oscillation maxima.

At the high NIR intensity, the Guinier amplitude drops to the background level within ~100 fs, indicating that most molecules within the x-ray focus disintegrate completely within that time span. The large contribution of small fragments and the presence of doubly charged carbon ions in the TOF spectrum (Fig. 3, B and F) support a violent Coulomb explosion. The radius (Fig. 2H) expands simultaneously with the decreasing amplitude (Fig. 2D) until the size of the corresponding scattering patterns has reached the geometrical detection limit imposed by the gap in the scattering detector (cf. fig. S4). Likewise, the simulated dynamics is dominated by fast Coulomb explosion, which manifests in a rapid reduction of the scattering amplitude and a corresponding rapid increase in the radius (Fig. 4, D and H). Details about our classical molecular dynamics (MD) calculations can be found in the “Theory” sections in Materials and Methods and the Supplementary Materials. The calculated scattering signals were analyzed with the same fitting procedure as the experimental data including and masked with the detector geometry.

In the $C_{60}$ dynamics, three cases can be identified: Bookended by the perturbative intensity, where amplitude and radius are both constant, and the high intensity, where the Guinier amplitude decreases simultaneously with the increase of the radius, the transition region of the low and intermediate intensities is characterized by a more complex expansion dynamics: The Guinier amplitudes drop with a delay and slower than the radii expand. While the radii expand to 50% of their respective maxima (marked as dashed blue lines in Fig. 2, F and G), the Guinier amplitudes drop by only 5% (Fig. 2B) and 10% (Fig. 2C) for the low and intermediate intensities, respectively. Thus, the differences in the dynamics of the radius expansion and the amplitude drop are intensity dependent: For the low intensity, the amplitude drops delayed by 430 fs (Fig. 2B) with respect to the radius expansion. Furthermore, the amplitude drop takes double the time [900 fs full width at half maximum (FWHM)] compared to the radius expansion (450 fs FWHM) (Fig. 3A). For the intermediate intensity, the delay of the amplitude drop reduces to 110 fs (Fig. 2C) and the expansion takes place within 140 fs, while the amplitude drops within 190 fs (Fig. 3A). The delayed drop of the amplitude with respect to the radius expansion indicates that the molecules undergo deformation and expansion before atomization sets in. In particular the small drop of the Guinier amplitude during the increase of the radius for the low intensity shows that the $C_{60}$ molecules can at most undergo a heavily asymmetric breakup into a large moiety and small fragments. Since the reduction of the amplitude scales linearly with the number of fragments for symmetric breakup (cf. section “Relation between Guinier amplitude and molecular fragmentation” in the Supplementary Materials), a decrease of the amplitude of less than a factor of two is only possible for asymmetric fragmentation. Thus, for the intermediate intensity where the amplitude drops by 50% after the initial radius expansion, the most symmetric breakup possible is in two equally large moieties. The more likely scenario to reach the reduction by a factor two is however the formation of a large moiety during the expansion, where the amplitude drops by only 10% at half rise and a further asymmetric breakup when reaching the maximal radius. Breaking bonds of the $C_{60}$ cage will produce initially large pieces, reducing the coherent scattering intensity only slightly and slowly before smaller fragments are formed, a hypothesis which would be in agreement with the experimental observation.

The intermediate intensity shows a remarkable additional feature: Following the fast expansion by a factor 1.5 within ~140 fs the radius shrinks again with a time constant of ~155 fs to 1.2 times the initial value (Fig. 2G and the “Fitting procedures” section in the Supplementary Materials). The decrease of the radius sets in when the Guinier amplitude has already decayed to half of its minimum. The strongly decreased Guinier amplitude during the radius drop indicates that a large fraction of molecules has already broken up. Thus, the reduced radius is a signature of scattering off small fragments. While is it likely that we excite Rydberg states, the observed MD can not be associated with the decay of these states since their lifetime is on the microsecond timescale (*32*).

The simulated Guinier amplitudes and radii (Fig. 4, B, C, F, and G) at large delays show good qualitative agreement with the experiment indicating that the asymptotic fragmentation patterns are well reproduced by our calculations. For the intermediate intensity, also the expansion of the radius with subsequent decrease is reproduced albeit with a smaller maximal radius. The delayed and slowed down drop of the Guinier amplitude with respect to the radius is however not present in the simulations. The most notable difference between the simulation and the measured data is the complete absence of the breathing mode oscillation in the experiment. The simulation for the low intensity predicts a substantial peak-to-peak amplitude of the oscillation of up to 20% for the Guinier amplitude and up to 10% for the radius. For the intermediate intensity, the simulated amplitude and radius (Fig. 4, C and G) are still clearly modulated by the breathing-mode oscillation but damped by fragmentation.

## DISCUSSION

We have explored the underlying reasons for the discrepancy between the measured and predicted $C_{60}$ expansion dynamics by adding ad-hoc terms to our classical theory model representing possible effects that may arise through the interplay of a coupled quantum many-body electron-ion system. As low-lying quantum states cannot be included in the classical model, we approached their contribution in a statistical fashion by adding a constant heating term per time step to our MD calculations. This can be considered to represent a global energy increase of the molecules through interaction of the free (laser-driven) electrons with the ones still quantum-mechanically bound and contributing to bonding and thus strongly couple to the positions of the ions/nuclei. By doing so, we find an earlier onset of Coulomb explosion and stronger damping of the oscillation (cf. “Theory” section in the Supplementary Materials). Depending on the heating conditions, the oscillation can be completely suppressed. Our coarse-grained approach to include multi-electron heating as a factor is a first step in understanding the coupling of electrons to nuclear motion in a complex quantum system, hereby studying its influence on the predicted breathing mode. By including this new mechanism in our modeling, we find that electronic heating on timescales faster than vibrations can suppress coherent internuclear motion. It may thus be the reason for the absence in the experiment with far-reaching implications for understanding and controlling in general complex systems exposed to lasers.

Most calculations have identified the excitation of the symmetric breathing (*18*) or the oblate-prolate (*13*, *20*) mode to be the crucial step to efficiently couple electronic excitation to nuclear degrees of freedom in $C_{60}$ , even under violent fragmentation conditions (*14*). Our experimental data shows no such periodic changes. Direct experimental evidence of the excitation of the breathing mode is sparse. It has been reported for a solid-state $C_{60}$ sample (*19*) and in a gas-phase experiment, in the latter case by applying shaped laser pulses optimized to create vibrationally hot fragments (*17*). The interpretation of other experimental results, like the complex fragmentation (*15*) or deformation (*21*) of $C_{60}$ molecules in strong fs laser pulses, has been based on the dominant excitation of one vibrational mode. In contrast to the majority of previous gas-phase experiments on $C_{60}$ , which applied techniques highly selective to specific charged fragments, we image the response of the full ensemble using x-ray scattering. Our results suggest that the vibrational coupling is not as selective as proposed by theory.

The poor agreement of simulation and experiment at small delays for the low and intermediate intensity shows that the current level of theory is not able to capture the complex multi-electron dynamics taking place during the interaction with a strong fs laser field. In particular, transient highly excited and ionized states, which decay via transfer processes faster than vibrational timescales, are not included in our calculations. Currently, a full quantum mechanical treatment including all intramolecular relaxation processes is technically not feasible. Thus, our combined experimental and theoretical study on strong field–ionized $C_{60}$ serves as ideal test ground to develop the necessary fundamental understanding of multi-electron dynamics in highly excited polyatomic molecular systems, which are needed to control chemical reactions with laser fields.

In conclusion, we have imaged the strong field–induced dynamics of an ensemble of gas-phase $C_{60}$ molecules using fs time-resolved x-ray diffraction. In the laser intensity transition regime between mostly stable and violently Coulomb-exploding molecules, the MD is governed by a fast expansion, which facilitates disintegration of the molecules afterward. This experiment demonstrates the feasibility of imaging laser-driven MD, which, as a typically nonresonant excitation process, triggers a plethora of different fragmentation processes and manipulates the system under investigation itself. Because of the low scattering cross sections of single molecules, our method required averaging over many shots and allows to retrieve only the structure of an ensemble of molecules. Similar ensemble-averaged data have been used to observe photochemical reactions using hard x-ray scattering where methods for structure retrieval are under development [e.g., (*23*–*26*)]. In contrast, e.g., x-ray–induced Coulomb-explosion imaging allows to retrieve structural information on a single-molecule level but is so far limited to smaller molecules and low laser intensities (*33*–*35*). Furthermore, x-ray imaging of strong field–driven MD as introduced here could be improved in the future by using nanofocusing technologies (*36*). Thus, this work lays the foundation to not only observe chemistry as it happens, but also to drive chemical reactions by intense laser fields using x-ray diffraction as a probe.

## MATERIALS AND METHODS

### Experiment

The experiments were conducted at the Linac Coherent Light Source (*37*) at the AMO endstation (*38*) with a photon energy of 1.8 keV ( $\lambda_{\text{FEL}}=0.69\;\text{nm}$ ) and intensities of $\sim 10^{16}\frac{W}{{\text{cm}}^{2}}$ . The collinear NIR ( $\lambda_{\text{NIR}}=800\;\text{nm}$ ) and x-ray beams were focused down to diameters of ~60 (FWHM) and ~20 μm (FWHM), respectively, and intersected the center of a $C_{60}$ beam (Fig. 1). The NIR intensity was varied between $2\times 10^{13}\frac{W}{{\text{cm}}^{2}}$ and $8\times 10^{14}\frac{W}{{\text{cm}}^{2}}$ . $C_{60}$ molecules were evaporated from an oven heated to 870 K, producing a sample density at the laser focus of ~10^11^ particles/cm^3^. After the interaction, both laser pulses passed through a gap in the large-area pnCCD detector that recorded the scattered x-rays. Scattering patterns were recorded as a function of the NIR-pump–x-ray-probe delay over a range of ±1 ps, where positive delays denote that the NIR pulses preceded the x-ray pulses (Fig. 2). Delay scans were repeated over 1 to 2 hours at 120 Hz repetition rate for each NIR intensity. The NIR intensity and the corresponding $C_{60}$ ionization and fragmentation patterns were characterized with an ion TOF spectrometer. An independent TOF-based NIR/x-ray cross-correlation measurement on $N_{2}$ yielded an instrument response function of 38 ± 3 fs (FWHM) with jitter correction using a time tool and was used to determine the time overlap (section “Experimental procedures” in the Supplementary Materials). The pulse durations of the x-ray and NIR laser pulses were both estimated to be 30 fs.

### Theory

The NIR-induced dynamics was calculated with classical MD simulations describing the laser-driven motion of electrons and ions (cf. the section “Theory” in the Supplementary Materials). These calculations are based on empirical two- and three-body forces (*39*) augmented by pairwise Coulomb interactions. Whereas the former account for the covalent bonds in $C_{60}$ , the latter are essential for describing multiply charged $C_{60}$ . With one active electron per atom, we include electron-electron collisions, electron screening of C ions, and Coulomb explosion of the ions. From the results of the MD calculations, x-ray scattering images were simulated using the experimental beam parameters and geometries and analyzed like the experimental data (Fig. 4). In addition, quantum dynamics simulations (cf. the section “Theory” in the Supplementary Materials) were carried out for the low intensity regime.

## Supplementary Materials

### The PDF file includes:

Supplementary Text Figs. S1 to S8 Legends for movies S1 to S4 References
