# Supplementary material for: Visualizing the strong field–induced molecular breakup of C60 via x-ray diffraction
Source: Sci Adv. 2025 Nov 21;11(47):eadz1900. doi: 10.1126/sciadv.adz1900 (PMC12637291; doi:10.1126/sciadv.adz1900)
Supplement: Supplementary file 1 — Supplementary Text Figs. S1 to S8 Legends for movies S1 to S4 References [file sciadv.adz1900_sm.pdf]

Science Advances
AAAS

# Supplementary Materials for

## Visualizing the strong field–induced molecular breakup of $C_{60}$ via x-ray diffraction

Kirsten Schnorr *et al.*

Corresponding author: Kirsten Schnorr, kirsten.schnorr@psi.ch; Sven Augustin, sven.augustin@psi.ch; Claus Peter Schulz, cps@mbi-berlin.de; Thomas Pfeifer, tpfeifer@mpi-hd.mpg.de

*Sci. Adv.* **11**, eadz1900 (2025)
DOI: 10.1126/sciadv.adz1900

**The PDF file includes:**

Supplementary Text
Figs. S1 to S8
Legends for movies S1 to S4
References

**Other Supplementary Material for this manuscript includes the following:**

Movies S1 to S4

# Supplementary Materials

## Experimental setup

Soft X-ray pulses with a central photon energy of 1.8 keV at a repetition rate of 120 Hz were generated by the Linac Coherent Light Source (LCLS) (*37*) at the Stanford Linear Accelerator Center (SLAC). Based on electron-bunch length measurements, the X-ray pulse duration was estimated to be 30 fs (FWHM). The $C_{60}$ pump-probe experiments were performed at the AMO (Atomic, Molecular and Optical science) endstation (*40*) using the LAMP instrument (*38*) configured in scattering geometry with a pair of pnCCD detectors (*41*) ($78\times74$ mm$^2$ full area, $75\times75$ µm pixel size, 3.2 cm gap) at a distance of 7 cm downstream of the interaction point. Using a pair of KB mirrors, the soft X-ray pulses were focused to a diameter of ∼20 µm (FWHM) reaching intensities of $\sim10^{16}\,\frac{\mathrm{W}}{\mathrm{cm}^2}$ in the interaction point assuming 20 % beamline transmission. Optical laser pulses with 800 nm central wavelength, 3 mJ pulse energy and ∼30 fs (FWHM) pulse duration were generated by the AMO Ti:sapphire laser system (*42*). The optical laser pulses were focused using an out-of-vacuum lens with a focal length of 1 m to a diameter of ∼60 µm (FWHM) and collinearly overlapped with the X-rays using an in-vacuum incoupling mirror mounted under 45° with a central hole allowing the X-rays to pass through. Spatial overlap of the NIR and X-ray beams was ensured by monitoring them on a frosted YAG crystal mounted on a diagnostics paddle that was inserted in the interaction point before and in between the pump-probe measurements. The NIR intensity was scanned between $2\times10^{13}\,\frac{\mathrm{W}}{\mathrm{cm}^2}$ and $8\times10^{14}\,\frac{\mathrm{W}}{\mathrm{cm}^2}$ using a polarizer–waveplate combination.

Both, the optical and X-ray beam were focused into a beam of gas-phase $C_{60}$ molecules, generated by an oven resistively heated to 870 K (*43*). $C_{60}$ powder (purchased from Sigma Aldrich with 99.9 % purity) was evaporated through a conically shaped copper tip with a 1 mm hole diameter reaching a target density of roughly $10^{11}\,\frac{\mathrm{particles}}{\mathrm{cm}^3}$ in the interaction point, located 5 mm below the tip. Simultaneous operation of the hot oven and the cryogenically cooled (−50 °C) pnCCD detectors was achieved by means of a three-layer heat shield around the oven.

Scattering images of an ensemble of $C_{60}$ molecules were recorded with the pnCCD detectors. In addition, time-of-flight (TOF) spectra were recorded alternating with the scattering measurements using a compact TOF spectrometer (*44, 45*). Details of the experimental setup are shown in Fig. S1.

The ion TOF spectrometer was designed with the purpose to serve as a compact detector for measuring charge state distributions to be used in combination with a large area scattering detector (*44*). As shown in Fig. S1, the TOF spectrometer was mounted on a linear stage to be able to retract it from the interaction region for operation with the pnCCD detector close to the oven and the catcher placed over the oven. Figure S1 shows the experimental setup in TOF configuration with the spectrometer in position for recording TOF spectra. The TOF spectrometer consists of a repeller and extractor

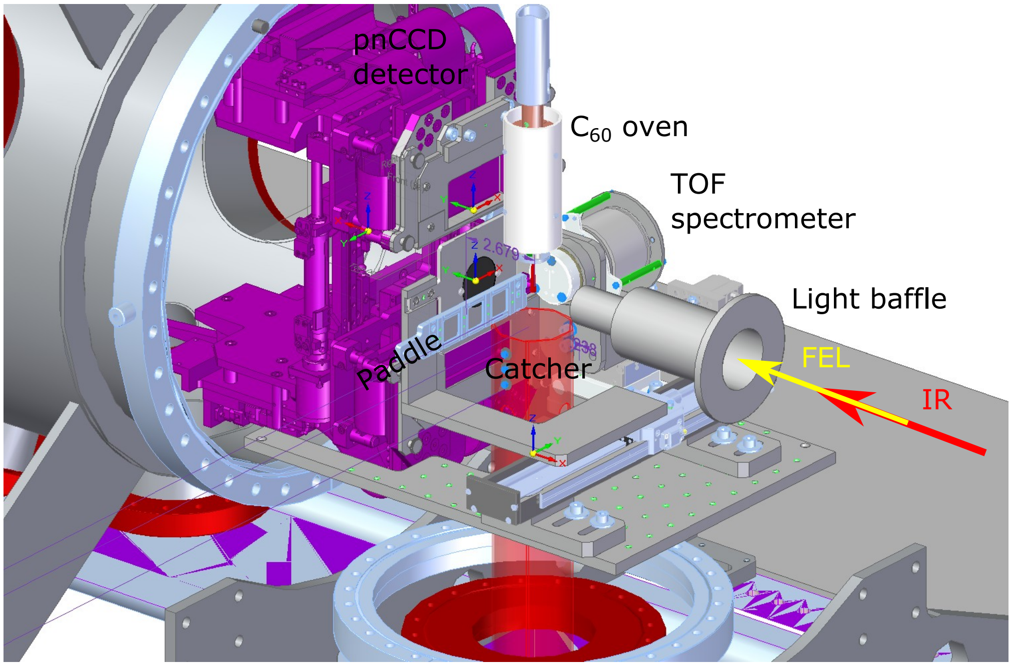


Figure S1: **Layout of the experimental setup.** The FEL and NIR beam arrive from the lower right, pass through a light baffle to suppress background scattering and interact with $C_{60}$ molecules injected by the resistively heated oven from the top. The oven is mounted on a xyz-manipulator for fine adjustment. Scattered photons are detected by the pnCCD detector in the back and the FEL and NIR beam pass through the adjustable central gap of the two detector halves. The $C_{60}$ molecular beam is dumped into a catcher, which is mounted on a xyz$\phi$-manipulator from the bottom to be able to completely cover the oven during operation of the cooled pnCCD detector. Alternatively – with the catcher lowered, as shown here – TOF spectra can be recorded with a compact TOF spectrometer, which is mounted on a linear translation stage to drive it into the interaction point. In this mode, the cooled pnCCDs are retracted to the back. Instead of the TOF spectrometer, a diagnostics paddle with a clear and a frosted YAG screen and a SMA "antenna" for establishing rough spatial and temporal overlap can be inserted into the interaction region.

electrode, with a separation of 40 mm between each other and the FEL passing through the center between the electrodes. The repeller was grounded and mounted on the diagnostics paddle while the extractor was kept at a potential of −900 V. The entrance to the 40 mm drift region was sealed off with a 1 mm width vertical slit and terminated with a mesh before the MCP. The TOF signal was detected with a commercial MCP/scintillator/photo multiplier combination (Photonis Bipolar TOF, APD BPTOF 25/6/5/12 D 60:1 MP EDR), as detailed in Ref. (*45*), and acquired with a commercial Aquiris digitizer. The front of the MCP facing the interaction region was supplied with a voltage of −2150 V, the back of the MCP with −1000 V, the scintillator with 2050 V and the photo multiplier with −800 V. Please note that for an ion kinetic energy of roughly 1 keV the detection efficiency for small fragments is larger than for large fragments (*28*).The polarization of both the FEL and the NIR beams was horizontal in the plane of the TOF spectrometer.

# Experimental procedures

NIR-pump–X-ray-probe spectra were recorded over a delay range of ±1 ps, where positive delays denote a preceding NIR pulse and negative delays preceding X-rays. The delay of the NIR pulse was continuously scanned during the measurement with a step size of 10 fs. For each NIR intensity, scans of 20 minutes were recorded and repeated multiple times. For each shot, the jitter between the NIR and X-ray pulses was characterized with a time tool based on the spectral encoding technique (*46*). Long-term timing drifts were measured and corrected every few hours by repeating a delay scan at the highest NIR intensity and monitoring the position of the sharp drop in scattering intensity due to disintegration of the $C_{60}$ molecules (cf. Fig. 2d). The spatial overlap of the NIR and the X-ray beam was fine tuned using the same signal, however at lower NIR intensities to avoid saturation effects.

The temporal overlap of the NIR and FEL pulses (referred to as time zero $t_0$) in the interaction point was determined in a separate delay-dependent TOF measurement on $N_2$ molecules. The method was previously applied and is described in detail in Ref. (*47*). Briefly, the yield of $N_2^{2+}$ ions divided by the yield of $N^{2+}$ ions, plotted as a function of the pump-probe delay, results in a step-function that indicates $t_0$ (cf. Fig. S2). The signal originates from $N_2^{2+}$ ions, produced by the absorption of one X-ray photon, which are dissociated by the NIR laser once it arrives later than the X-rays, resulting in a depleted number of bound dications and an increased number of $N^{2+}$ ions. Each shot is jitter-corrected with the time-tool information. In order to determine time zero, the delay-dependent step function is fitted with a generalized error function

$$e(t) = a \cdot \mathrm{erf}\left(\frac{t - t_0}{\sigma}\right) + c \qquad (5)$$

with the fit parameters: amplitude $a$, constant offset $c$, $t_0$, and $\sigma$, where FWHM $= 2 \cdot \sqrt{\ln 2} \cdot \sigma$ corresponds to the instrument response function. Multiple scans were repeated,

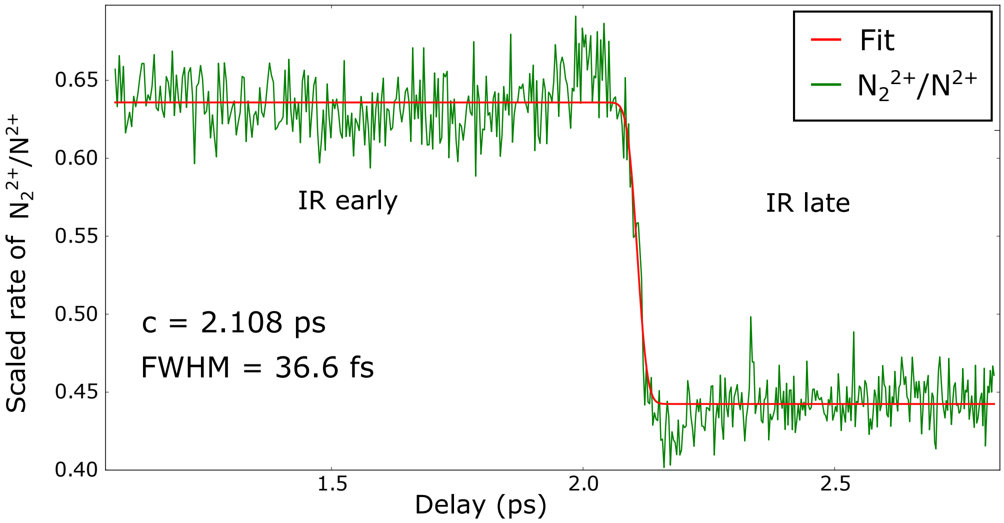


Figure S2: **Delay-dependent yield of molecular nitrogen dications $N_2^{2+}$, divided by the yield of dissociated $N^{2+}$ ions.** The fit of the error function yields $t_0$ and an instrument response function of 37 fs (FWHM). The delays were jitter-corrected using a time tool.

yielding an instrument response function of $38 \pm 3$ fs (FWHM).

# Detector calibration

The scattering detector calibration was performed in four steps, as illustrated in Fig. S3. The raw image, shown in Fig. S3a, contains all detected photons. The photon-energy distribution in analog-to-digital units (ADU) is shown in Fig. S3e. A cut-out around the photon energy of 1.8 keV of elastically scattered photons is applied, resulting in Fig. S3b. The same energy range was used for all recorded data. Some malfunctioning areas on the detector require a gain correction which was performed by scaling the gain in these areas to match the gain to the rest of the detector, as illustrated in Fig. S3f,g. A gain-corrected detector image is shown in Fig. S3c. Since the gain was slowly drifting over time, the gain correction was repeated for every scan. Furthermore, all images contain background scattering from beamline components, which was minimized in every shift but could not be completely avoided. A new background image without the NIR laser and no target, such as Fig. S3h, was recorded at the beginning of each shift after beamline stray light optimization and subtracted from the gain corrected detector image for each scan.

We detected on average 120 scattered photons per shot, with $\sim$10 of these photons stemming from background.

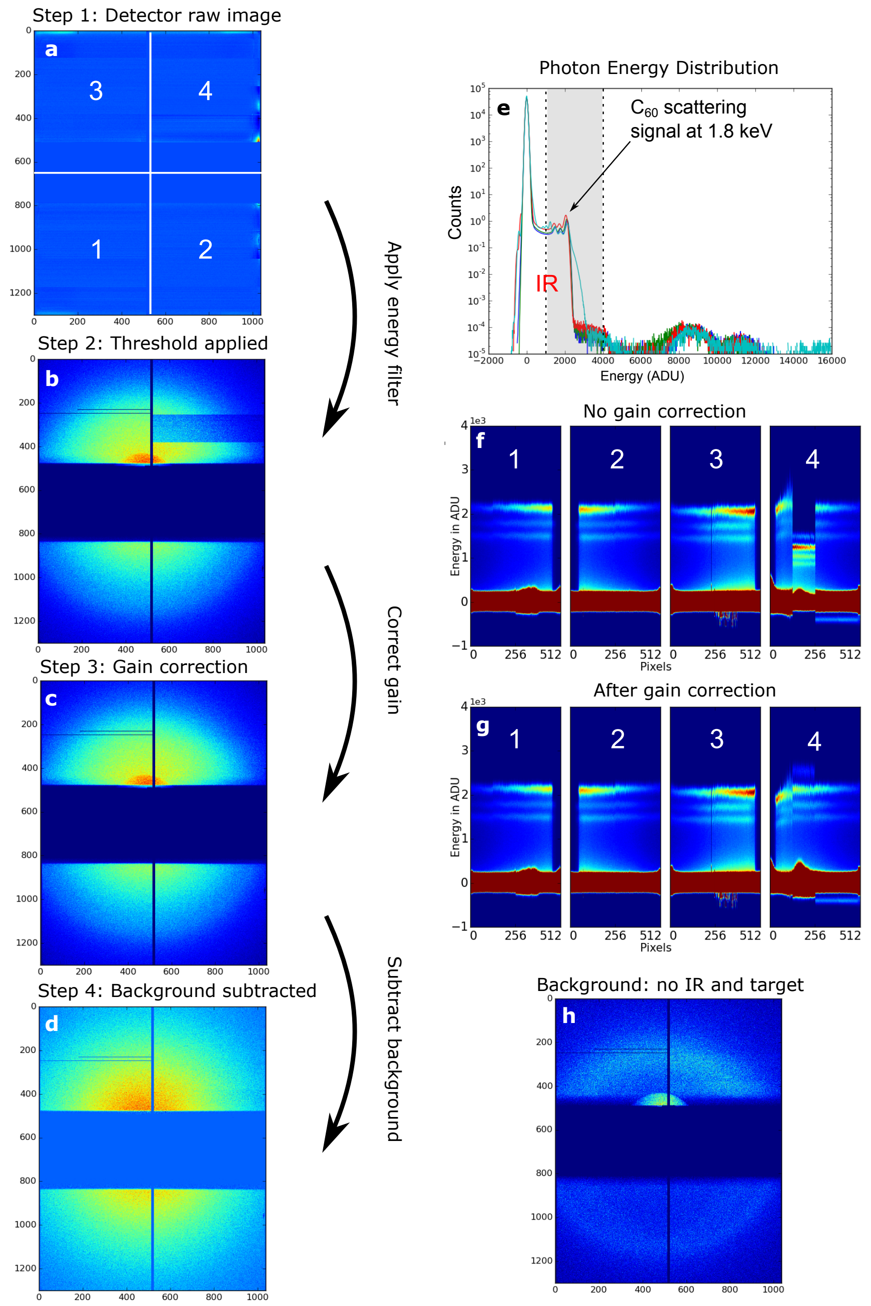


Figure S3: **Applied sequence of detector calibration steps.** (**a**) raw detector image, sectors are labeled with numbers 1–4; (**b**) detector image of selected photons with energies around the elastic scattering photon energy, shown in (**e**); (**c**) detector image after gain correction of sector 4, comparison before and after shown in (**f**) and (**g**) respectively; (**d**) detector image after background subtraction; (**h**) background recorded with no optical laser and without target.

# Guinier fit

In order to relate the changes in the widths of the scattering images with the structural changes of the $C_{60}$ molecules, we perform Guinier fits on the recorded images (following the description in Sec. 2.1.2.6 of Ref. (*27*)). We extract the normalization of the delay-dependent radii (cf. Fig. 2e-h) from Guinier fits to static X-ray diffraction data, i.e., from unperturbed $C_{60}$ molecules.

The scattering intensity $I(q, R)$ for a spherical object is given by:

$$I(q, R) = I_{\mathrm{e}}(q)\, F^2(q, R), \tag{6}$$

$$F(q, R) \stackrel{!}{=} F_{\mathrm{Guinier}}(q, R) = \underbrace{V_{\mathrm{sph}}(R)\,\rho_{\mathrm{e}}}_{N} \left(3\,\frac{\sin(qR) - qR\,\cos(qR)}{(qR)^3}\right), \tag{7}$$

where $q = 4\pi \sin(\theta/2)/\lambda$ is the momentum transfer, $\theta$ the scattering angle, $\lambda$ the wavelength, $R$ the radius of the sphere, $I_{\mathrm{e}}(q)$ the intensity scattered by a free electron, $V_{\mathrm{sph}}(R) = \frac{4}{3}\pi R^3$ the volume of the target, and $\rho_{\mathrm{e}} = \frac{N}{V_{\mathrm{sph}}(R)}$ the average electronic scatterer density with $N$ being the number of scatterers.

For an ellipsoidal target, R is replaced by

$$r(R_{\mathrm{pol}}, R_{\mathrm{equ}}, \theta) = \sqrt{R_{\mathrm{equ}}^2\, \sin^2\theta + R_{\mathrm{pol}}^2\, \cos^2\theta} \tag{8}$$

and the volume $V_{\mathrm{sph}}(R)$ becomes $V_{\mathrm{elip}}(R_{\mathrm{pol}}, R_{\mathrm{equ}}) = \frac{4}{3}\pi\, R_{\mathrm{pol}}\, R_{\mathrm{equ}}^2$.

For the fit of the scattering images, we added a constant background and re-arranged the function to

$$I(q, R_{\mathrm{pol}}, R_{\mathrm{equ}}) = A(q, R_{\mathrm{pol}}, R_{\mathrm{equ}}) \left(3\,\frac{\sin(qR) - qR\,\cos(qR)}{(qR)^3}\right)^2 + \mathrm{const}, \tag{9}$$

$$A(q, R_{\mathrm{pol}}, R_{\mathrm{equ}}) = I_{\mathrm{e}}(q)\, V_{\mathrm{elip}}^2(R_{\mathrm{pol}}, R_{\mathrm{equ}})\, \rho_{\mathrm{e}}^2 = I_{\mathrm{e}}(q) N^2, \tag{10}$$

with $R = r(R_{\mathrm{pol}}, R_{\mathrm{equ}}, \theta)$ as in Eq. 8. $I_{\mathrm{e}}(q)$ is assumed to be constant in $\theta$. Thus, $A(q, R_{\mathrm{pol}}, R_{\mathrm{equ}})$ becomes $A(R_{\mathrm{pol}}, R_{\mathrm{equ}})$, i.e., independent of $\theta$. As $A$ is now only dependent on fit parameters, it can simply be a constant fit parameter for the whole detector plane. Since $A$ is proportional to the number of scatterers squared, it is a measure of the degree of fragmentation and ionization of the $C_{60}$ molecules.

In the calculation of the fit function, we take the detector shape into account. Specifically, we calculate an intensity for each 3D pixel position, hence this includes the $\cos^3\theta$ distortion.

Figure S4 shows the detector images from Fig. 1 and illustrates the performance of the Guinier fit. Four different delay ranges at the intermediate NIR intensity are selected to showcase different scattering scenarios:

- delay $< 0\,\text{fs}$: intact molecules with highest photon yield. The $C_{60}$ ground-state radii in Fig. 2 and Fig. 4 are normalized to the mean of the fitted Guinier radius for negative delays.
- delay $\sim 0\,\text{fs}$: expanding molecules with slightly reduced photon yield
- delay $> 0\,\text{fs}$: Coulomb-exploding molecules with clearly reduced photon yield
- delay $\gg 0\,\text{fs}$: disintegrated molecules with lowest photon yield

The panels in Fig. S4 are grouped according to the four aforementioned delay ranges. Each subfigure (a) shows the experimental raw detector image with $4\times4$ pixels binned together. The corresponding ellipsoidal Guinier fit results with detector mask are shown in subfigures (b). Please note that reported Guinier fit results always stem from ellipsoidal Guinier fits. The projections on the y-axis of subfigures (a) and (b) are shown in blue and orange respectively in subfigures (c). The results of the Guinier fits without detector mask are shown in subfigures (d). The raw detector images with gaps filled from the fit are shown in subfigures (e). The projections on the y-axis of subfigures (d) and (e) are shown in orange and blue respectively in subfigures (f). The ensemble-averaged Guinier radii shown in Fig. 2 and Fig. 4 correspond to the fit results from the y-projection. The y-direction was chosen because the detector covers a larger area. The fit results in x-direction agree within error bars.

## Relation between Guinier amplitude and molecular fragmentation

The Guinier amplitude is proportional to the squared number of scatterers in each moiety ($N^2$), as shown in Eq. 10. We denote the number of scatterers for an intact $C_{60}$ molecule as $N_0$. If we assume a break-up into $m$ equally large fragments with $N_m = \frac{N_0}{m}$, the ratio of Guinier amplitudes $A_0$ for an intact molecule and $A_m$ for $m$ fragments is

$$\frac{A_0}{A_m} = \frac{N_0^2}{\sum_{i=1}^{m} N_i^2} = \frac{N_0^2}{m\left(\frac{N_0}{m}\right)^2} = m. \tag{11}$$

Thus, the Guinier amplitude decreases linearly with the number of fragments under the assumption that fragments are (roughly) equally large. For very asymmetric break-up into a large moiety and small fragments, the Guinier amplitude drops slower with the number of fragments because the term $N_{\text{large}}^2$ for a large fragment dominates the denumerator.

For the direct removal of $k$ electrons from a structural intact $C_{60}$ molecule through photoionization by the NIR laser, the Guinier amplitude drops to $(N_0 - k)^2$.

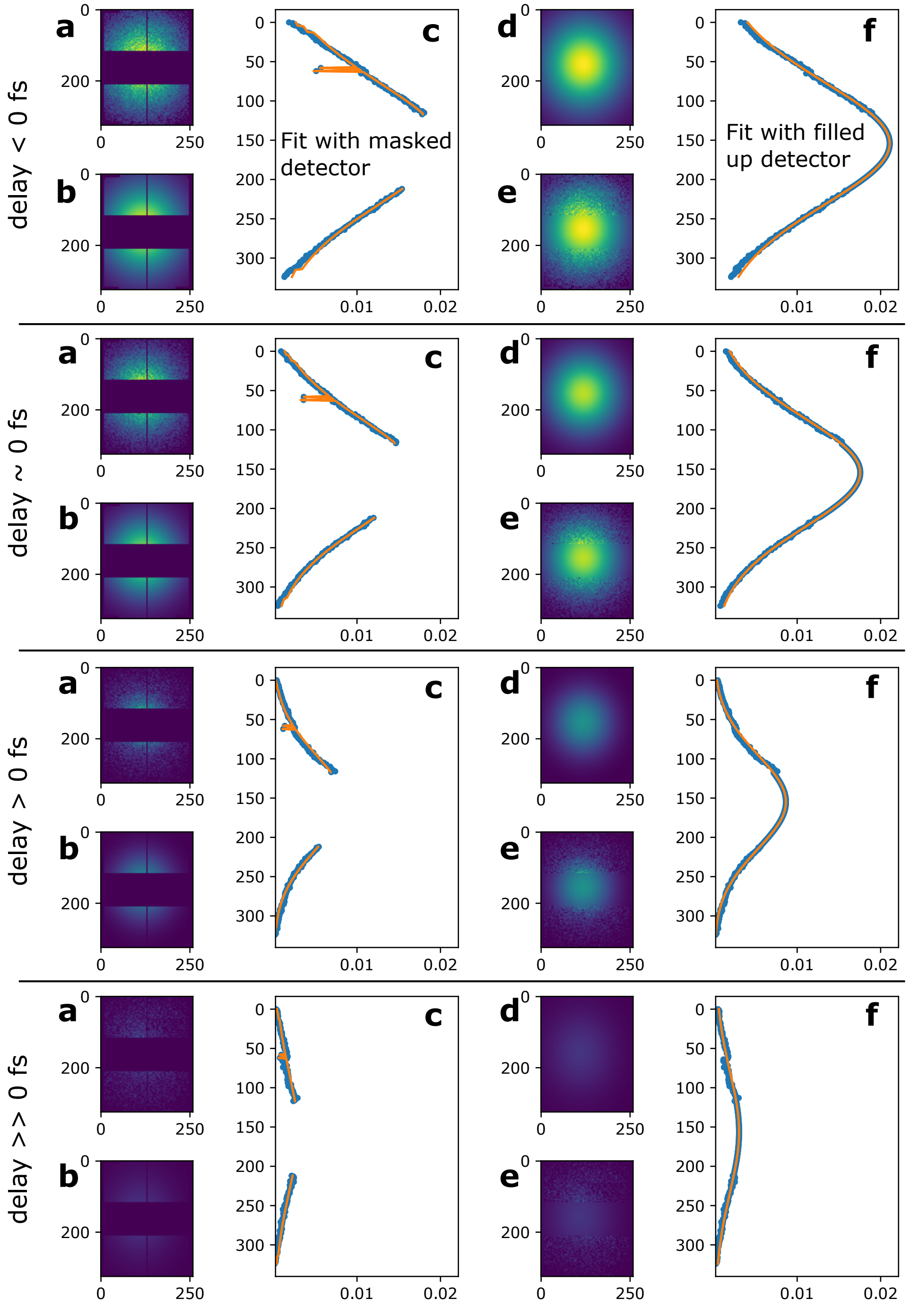


Figure S4: **Ellipsoidal Guinier fits at the intermediate NIR intensity for four different delays (from top to bottom), grouped in panels respectively.** **a**, raw detector image with 4×4 pixel binning; **b**, fit result with detector mask; **c**, projections on y axis of (**a**) in blue and (**b**) in orange; **d**, fit result without mask; **e**, image (**a**) with gaps filled from fit and **f**, projections on y axis of (**d**) in orange and (**e**) in blue.

# Uncertainty estimation

The error bands in Fig. 2 are calculated by selecting the first 350 fs (35 bins $\times$ 10 fs) of each delay scan and determining the standard deviation thereof. Since these delays lie in the negative range before the molecules show any dynamical response, the fluctuations represent the noise of the data.

# Fitting procedures

The fitting procedure to extract time scales for the dynamics of the ensemble-averaged radius at the intermediate intensity are illustrated in Fig. S5. The expansion dynamics, represented by the rising edge in Fig. S5 is fitted with an error function $r(t)$. The decay of the radius is fitted with an exponential decay function $d(t)$. The complete fitting function is the product of $r(t)$ and $d(t)$, convoluted with a Gaussian function with a width corresponding to the instrument response function. The fit yields a rise time of $140 \pm 5$ fs (FWHM) and an exponential decay time $\tau = 155 \pm 10$ fs.

$$r(t) = \mathrm{erf}\left(\frac{t - t_0}{\sigma}\right) \tag{12}$$

with $\mathrm{FWHM} = 2 \cdot \sqrt{\ln 2} \cdot \sigma$

$$d(t) = \exp\left(-\frac{t - t_0}{\tau}\right) + c \tag{13}$$

Figure 3a shows the decrease of the delay-dependent scattering amplitudes for different IR intensities. For each IR intensity, multiple delay scans were recorded and each of them fitted with an error function (cf. Eq. 7). The plotted data points represent the averaged fitted FWHM of the scattering amplitude steps, as shown for the low, intermediate and high NIR intensity and a single data set in Fig. 2b-d. The given error bars represent the standard error.

## Influence of X-ray pulses

We neglect perturbation by the X-rays (i.e., ionization and break-up induced thereof within the X-ray pulse duration) in the experimental interpretation and do not include the interaction of the probe pulse into the simulations. This assumption is based on the results of previous work which has explicitly studied the response of $C_{60}$ to intense XFEL pulses. Both, the fragmentation of $C_{60}$ in single intense XFEL pulses (*48*) and the X-ray induced evolution of the break-up was studied using an X-ray pump-probe scheme (*12*). The latter publication comes to the conclusion that the atomic displacement for up to 30 fs after the peak of the X-ray pulse does not exceed 2 Å. We have compared the pulse parameters reported in Ref. (*12*) with the current study and conclude that the amount

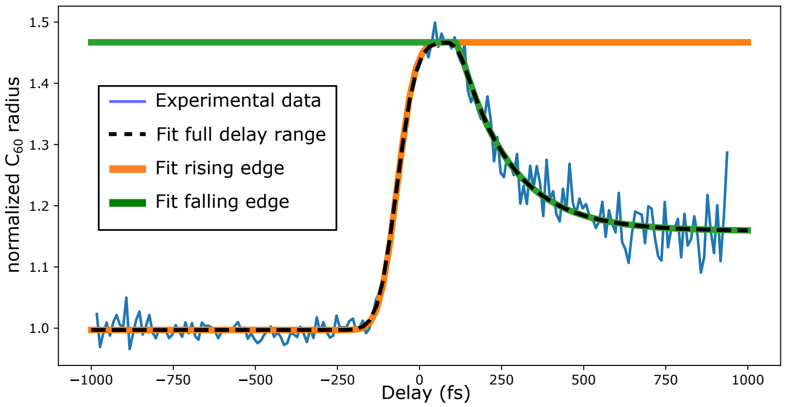


Figure S5: **Fit procedure for Fig. 2g.** The experimental data (blue solid line) is shown together with the fit over the full delay range (black dashed line). The fit function takes into account the rising edge, which is fitted with an error function (solid orange line), the falling edge, which is fitted with an exponential decay (solid green line), and both folded with a Gaussian function representing the instrument response function. The fit yields a rise time of $140 \pm 5$ fs (FWHM) of the error function and an exponential decay time $\tau = 155 \pm 10$ fs.

of photons absorbed per molecule should be about a factor of 3 lower in the present experiment. Thus, the displacement of the atoms within our 30 fs pulses can be safely neglected. Based on Ref. (*48*), we can estimate the average amount of absorbed X-ray photons per pulse to about 5, which should result in a loss of in average less than 10 electrons over the pulse from photoionization and Auger-Meitner decay. The effect on the scattering cross section (5 parts in 360) is therefore minor.

# Theory

## Classical molecular dynamics simulations

The process of laser strong-field excited/ionized $C_{60}$ molecules is calculated by means of classical molecular dynamics (MD) similar to the ones applied to clusters (*49*) and fullerenes (*12*) previously. This is justified by the fact that at these rather high intensities of the NIR laser pulses low-lying electronic states do not play a role but many highly-excited electrons are created. This situation can be considered as a nano-plasma. Important processes like electron-ion or electron-electron collisions or electron transfer (hopping) are all contained in the description, albeit on a classical level. A (TD)DFT description is not only out of reach since (i) we have to treat simultaneously many electrons bound to the $C_{60}$ atoms as well as continuum electrons quivering in the laser field over large distances, and (ii) we have to perform a proper focal averaging over many intensities.

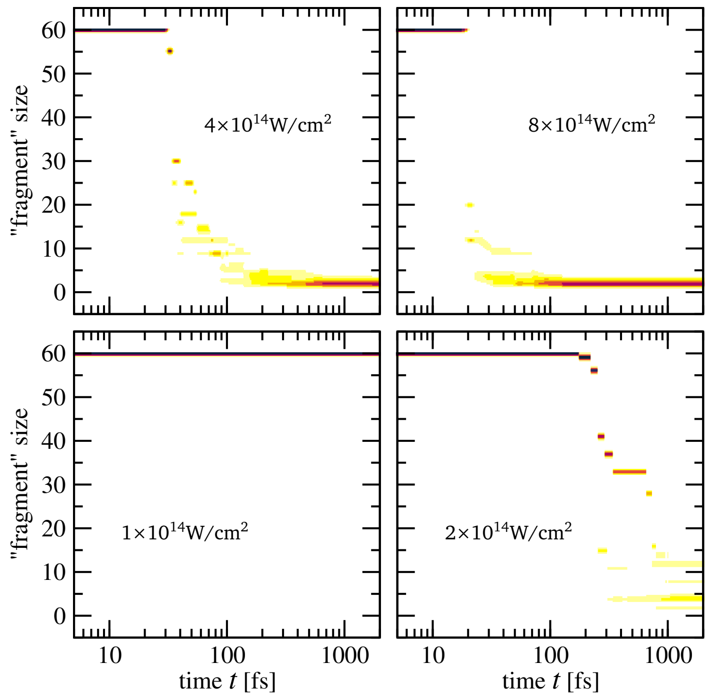


Figure S6: **Time-dependent fragment-size distribution of $C_{60}$ for four different laser intensities, calculated using our classical dynamics simulations.** The peak of the NIR pulse is at time $t = 0$ fs.

It would also completely fail to account for electron-electron collisions, which are crucial in a dense, quickly thermalizing nano-plasma like this.

Our calculations are based on standard empirical short-range two- and three-body forces (*39*) developed for the description of systems with covalent bonds. As a test, we have reproduced melting calculations (*50*), which were based on different forces. The initial temperature of the $C_{60}$ in the simulations was set to 800 K.

A new feature of our simulation is the inclusion of pairwise Coulomb interactions between electrons and carbon ions, which is required to account for (multiple) ionization of $C_{60}$. Therewith the equations of motions read

$$\ddot{\mathbf{r}}_j = \sum_{j' \neq j} \mathbf{f}_\alpha(\mathbf{r}_j, \mathbf{r}_{j'}) - \sum_J \mathbf{f}_\alpha(\mathbf{r}_j, \mathbf{R}_J) - \mathcal{E}(t) \tag{14a}$$

$$M\ddot{\mathbf{R}}_J = \sum_{J' \neq J} \mathbf{f}_\alpha(\mathbf{R}_J, \mathbf{R}_{J'}) - \sum_j \mathbf{f}_\alpha(\mathbf{R}_{\{J'\}}, \mathbf{r}_j) + \mathbf{F}_J(\mathbf{R}_J) + \mathcal{E}(t) \tag{14b}$$

$$\mathbf{f}_\alpha(\mathbf{x}, \mathbf{y}) \equiv \frac{\mathbf{x} - \mathbf{y}}{|[\mathbf{x} - \mathbf{y}]^2 + \alpha^2|^{3/2}} \tag{14c}$$

$$\mathcal{E}(t) \equiv \mathbf{e}_z \, \mathrm{e}^{-2\ln 2\, t^2/T^2} \sin(\omega t), \tag{14d}$$

with capital ($M$, $\mathbf{R}$, $J$) and small ($\mathbf{r}$, $j$) letters denoting ions and electrons, respectively. We treat one electron per atom, i. e. all indices run from 1 to 60. The Coulomb interaction $\mathbf{f}_\alpha$ is smoothed in order to prevent artificial auto-ionization as it is done routinely in MD calculations of laser-cluster interactions (*49*). We have chosen as smoothing parameter $\alpha = 3.58\,\mathrm{a}_0$, which guarantees that the ionization potential $\mathrm{IP}_{\mathrm{C}_{60}} = 7.6\,\mathrm{eV}$ is correctly reproduced. We use the standard expression for the Tersoff force $\mathbf{F}_J$ (*39*). We do not adjust the Tersoff force when the $C_{60}$ becomes charged. The driving laser pulse $\mathcal{E}(t)$ has a Gaussian envelope with a FWHM duration $T$ and the laser frequency $\omega$ defined by the wavelength $\lambda = 800\,\mathrm{nm}$. The low number of particles does not require any sophisticated propagation scheme.

We follow the positions of all electron and ions over time according to the Eqs. (14) and calculate scattering images with

$$P_\delta(k_x, k_y) = \int \mathrm{d}t\, \mathrm{e}^{-4\ln 2\,[t-\delta_\mathrm{x}]^2/T_\mathrm{x}^2} \left| p(k) \sum\nolimits_J \mathrm{e}^{\mathrm{i}\mathbf{k}\cdot\mathbf{R}_J(t)} \right|^2 \tag{15a}$$

$$\mathbf{k} = \{k_x, k_y, 0\}, \qquad k = |\mathbf{k}|, \tag{15b}$$

with the atomic structure factor $p(k)$, which is not altered for charged $C_{60}$. Note that we average in Eq. (15a) over the time $t$ weighted with a Gaussian representing the X-ray pulse with a FWHM of $T_\mathrm{x}$ and a delay with respect to the driving NIR laser of $\delta_\mathrm{x}$. The time-dependent fragment-size distribution is plotted in Fig. S6. Notably, the break-up into smaller fragments accelerates with the laser intensity, which is in line with the experimental findings.

## Additional heating

The calculation presented above does not reproduce the abrupt crossover from long-term stability to direct fragmentation, as observed in the experiment. One possible reason might be that the strong NIR pulse interacts with the core electrons leading to additional heating of the $C_{60}$ backbone (*11*). To account for this heating we use a simple ad-hoc term in our calculation: In each time step all carbon atoms are kicked along their instantaneous velocity. The total intensity-dependent heating energy is defined as $\Gamma(t) = \frac{I(t)}{10^{14}\mathrm{W/cm}^2}\Gamma_0$ with $I(t)$ the instantaneous intensity and $\Gamma_0 = 40\,\mathrm{eV/fs}$. Figure S7 shows the result of the this calculation where one sees indeed an abrupt unset of the increase of the $C_{60}$ radius indicating the onset of fragmentation.

## Quantum dynamics simulations

In the quantum dynamics simulations, the neutral ground state (GS) of $C_{60}$ is photoexcited and photoionized with a few-cycle NIR pulse centered at $t_0$, with a wavelength of $\lambda = 720\,\mathrm{nm}$ ($\omega = \frac{2\pi v}{\lambda}$) and a CEP $\phi = 0$. The peak intensity is $5.6 \times 10^{13}\,\frac{\mathrm{W}}{\mathrm{cm}^2}$, which gives a maximum electric field $E_0$ of 0.04 au. The pulse is linearly polarized in the $y$ direction,

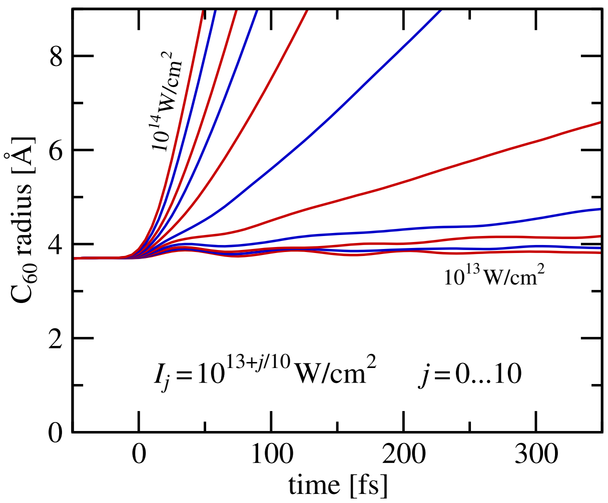


Figure S7: **Effect of the intensity-dependent heating.** The radius of $C_{60}$ is shown as a function of time $t$ for various intensities $I_j$ specified in the figure. The plotted radius is defined as $R(t) = \frac{1}{60}\sum_{A=1}^{60} |\mathbf{R}_A(t)|$ with $\mathbf{R}_A(t)$ being the position of atom $A$ in the center-of-mass frame at time $t$ as obtained with the MD calculation.

$\boldsymbol{e} = \boldsymbol{e}_y$. The analytical form of the time profile of the electric field is derived from the derivative of the vector potential $\boldsymbol{A}(t)$, $\boldsymbol{E}(t) = -\frac{d\boldsymbol{A}(t)}{dt}$ to ensure that there is no source term (*51*):

$$\boldsymbol{E}(t) = \boldsymbol{e}\, E_0 \exp\left(\frac{-(t-t_0)^2}{2\sigma^2}\right)\left[\cos(\omega t + \phi) - \frac{2(t-t_0)\sin(\omega t + \phi)}{\omega\sigma^2}\right] \tag{16}$$

$\boldsymbol{A}(t)$ has a Gaussian envelope with a $\sigma$ of 1.36 fs, (FWHM $= 2\sqrt{2\ln 2}\sigma = 3.2$ fs). The time-dependent Schrödinger equation (TDSE), $i\hbar\frac{d\psi(t)}{dt} = H(t)\psi(t)$, is solved for the electronic wave function $\psi(t)$ at the equilibrium geometry of the neutral GS. The initial state, $\psi(0)$, is the ground electronic state. The coupling with the electric field that leads to photoexcitation and photoionization is included explicitly in the electronic Hamiltonian in the dipole approximation, $\boldsymbol{H}(t) = \boldsymbol{H}_0^{\mathrm{elec}}(\boldsymbol{r}) - \boldsymbol{E}(t)\cdot\boldsymbol{\mu}$, where $\boldsymbol{r}$ stands for the electronic coordinates and $\boldsymbol{\mu}$ is the electronic dipole operator. The TDSE is solved using a coupled-equation scheme as described in Refs. (*52*) and (*53*) based on the partitioning technique in a bound subspace $\mathbf{Q}$ and an ionized subspace $\mathbf{P}$. The ground state and 406 excited states converging to the ionization potential are included in the bound subspace $\mathbf{Q}$. The electronic structure of the 500 lowest excited states of $C_{60}$ has been computed in TD-DFT with the long range corrected CAM-B3LYP functional (*54*) and a 6-31+G(d) basis set augmented by 6 s, p and d additional diffuse functions centered in the middle of the $C_{60}$ cage (*55*). Very diffuse basis functions were added in order to describe the highly excited Rydberg and SAMO (Super-Atom-Molecular-Orbitals) states below the ionization potential (IP). The ionized subspace $\mathbf{P}$ is spanned on a basis of ionized states built from

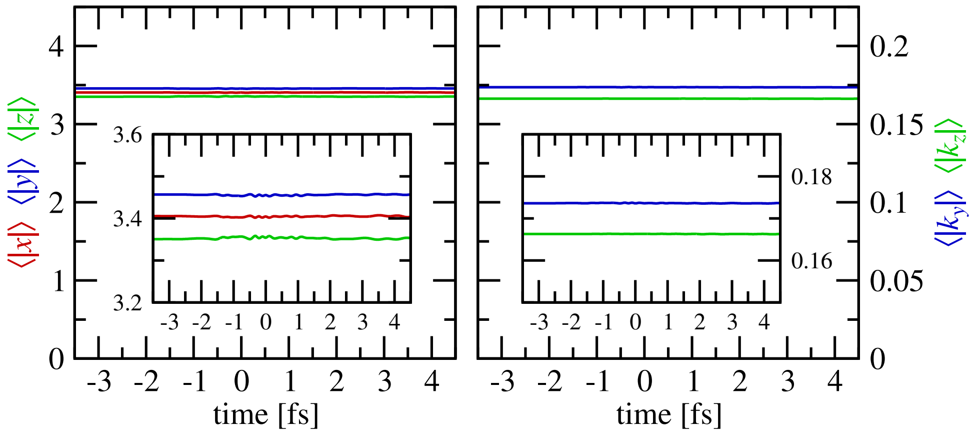


Figure S8: **Delay-dependent scattering signal extracted from quantum calculations.** The average absolute values of $x$, $y$ and $z$ of the electron density is shown in the left panel. The $k_y$- and $k_z$-components in the detector plane are shown in the right panel.

the antisymmetrized product of the field-free ground state of the cation $\Psi_{\text{cat}}$ and the wavefunction of the ionized electron $\chi_k$ which is an orthogonalized plane wave (*56, 57*) with a wave vector $\mathbf{k}$. The ground state of the cation is five times degenerate. For each cationic state, an ionization continuum discretized in energy and angle is associated. The momentum of the ionized electron varies between 0 and 2 au with a stepsize of 0.01 au and a set of 80 solid angle values is associated to each momentum value. Therefore, the ionized subspace is spanned on a basis of 80'000 states.

The results obtained from the quantum calculations are shown in Fig. S8. The laser-induced modulation in the electron density under these intensity conditions are too small to be visible in scattering images. Longer laser pulses in this regime would further ionize the system and thus infringe the validity of using the 5 continua of a singly ionized $C_{60}$ states, but massively blow up this hyper-continuum state space (exponentially with the number of ionized electrons). Intensities higher than $5.6 \times 10^{13}\,\frac{\text{W}}{\text{cm}^2}$ cannot currently be treated because they would require a prohibitive amount of states in the many-body continuum subspace. For intensities higher than $\sim 5 \times 10^{14}\,\frac{\text{W}}{\text{cm}^2}$, one would even need to go beyond the dipole approximation. The results using the $5.6 \times 10^{13}\,\frac{\text{W}}{\text{cm}^2}$ field strength factually prove that the complex electronic dynamics arising with optical laser intensities higher than $5.6 \times 10^{13}\,\frac{\text{W}}{\text{cm}^2}$ cannot be captured by fully quantum calculations.

# Supplemental movies

We provide movies of the classical MD simulations for the four NIR intensities displayed in Fig. 4. They show the scattering images of a single $C_{60}$ molecule (left) with the dynamics of the corresponding molecule (middle) and the ensemble averaged scattering pattern

(right). The following four files for perturbative, low, intermediate and high intensities, respectively, are available as supplemental movies:

**Caption for i2.0e13-l800-d45-dx25.mp4:** Movie 1 “Perturbative Intensity Regime” – Time-dependent response and scattering images of laser-excited single (left) and ensemble-averaged (right) $C_{60}$ molecules at a laser intensity of $2 \times 10^{13}\,\frac{\mathrm{W}}{\mathrm{cm}^2}$.

**Caption for i1.1e14-l800-d45-dx25.mp4:** Movie 2 “Low Intensity Regime” – Time-dependent response and scattering images of laser-excited single (left) and ensemble-averaged (right) $C_{60}$ molecules at a laser intensity of $1.1 \times 10^{14}\,\frac{\mathrm{W}}{\mathrm{cm}^2}$.

**Caption for i2.5e14-l800-d45-dx25.mp4:** Movie 3 “Intermediate Intensity Regime” – Time-dependent response and scattering images of laser-excited single (left) and ensemble-averaged (right) $C_{60}$ molecules at a laser intensity of $2.5 \times 10^{14}\,\frac{\mathrm{W}}{\mathrm{cm}^2}$.

**Caption for i8.0e14-l800-d45-dx25.mp4:** Movie 4 “High Intensity Regime” – Time-dependent response and scattering images of laser-excited single (left) and ensemble-averaged (right) $C_{60}$ molecules at a laser intensity of $8 \times 10^{14}\,\frac{\mathrm{W}}{\mathrm{cm}^2}$.

## REFERENCES AND NOTES


1. A. D. Bandrauk, *Molecules in Laser Fields* (Springer Netherlands, Dordrecht, 1995), pp. 131–150.

2. B. J. Sussman, D. Townsend, M. Y. Ivanov, A. Stolow, Dynamic stark control of photochemical processes. *Science* **314**, 278 (2006).

3. P. H. Bucksbaum, A. Zavriyev, H. G. Muller, D. W. Schumacher, Softening of the $H_2^+$ molecular bond in intense laser fields. *Phys. Rev. Lett.* **64**, 1883 (1990).

4. L. J. Frasinski, J. H. Posthumus, J. Plumridge, K. Codling, P. F. Taday, A. J. Langley, Manipulation of bond hardening in $H_2^+$ by chirping of intense femtosecond laser pulses. *Phys. Rev. Lett.* **83**, 3625 (1999).

5. M. E. Corrales, J. González-Vázquez, G. Balerdi, I. R. Solá, R. de Nalda, L. Bañares, Control of ultrafast molecular photodissociation by laser-field-induced potentials. *Nat. Chem.* **6**, 785 (2014).

6. J. Kim, H. Tao, J. L. White, V. S. Petrović, T. J. Martinez, P. H. Bucksbaum, Control of 1,3-cyclohexadiene photoisomerization using light-induced conical intersections. *J. Phys. Chem. A* **116**, 2758–2763 (2012).

7. H. W. Kroto, J. R. Heath, S. C. O'Brien, R. F. Curl, R. E. Smalley, $C_{60}$: Buckminsterfullerene. *Nature* **318**, 162 EP (1985).

8. W. Krätschmer, L. D. Lamb, K. Fostiropoulos, D. R. Huffman, Solid $C_{60}$: A new form of carbon. *Nature* **347**, 354–358 (1990).

9. F. Lépine, Multiscale dynamics of $C_{60}$ from attosecond to statistical physics. *J. Phys. B At. Mol. Opt. Phys.* **48**, 122002 (2015).

10. E. E. B. Campbell, K. Hansen, K. Hoffmann, G. Korn, M. Tchaplyguine, M. Wittmann, I. V. Hertel, From above threshold ionization to statistical electron emission: The laser pulse-duration dependence of $C_{60}$ photoelectron spectra. *Phys. Rev. Lett.* **84**, 2128 (2000).

11. I. V. Hertel, T. Laarmann, C. P. Schulz, Ultrafast excitation, ionization, and fragmentation of $C_{60}$. *Adv. Atom. Mol. Optic. Phys.* **50**, 219–286 (2005).

12. N. Berrah, A. Sanchez-Gonzalez, Z. Jurek, R. Obaid, H. Xiong, R. J. Squibb, T. Osipov, A. Lutman, L. Fang, T. Barillot, J. D. Bozek, J. Cryan, T. J. A. Wolf, D. Rolles, R. Coffee, K. Schnorr, S. Augustin, H. Fukuzawa, K. Motomura, N. Niebuhr, L. J. Frasinski, R. Feifel, C. P. Schulz, K. Toyota, S. K. Son, K. Ueda, T. Pfeifer, J. P. Marangos, R. Santra, Femtosecond-resolved observation of the fragmentation of buckminsterfullerene following x-ray multiphoton ionization. *Nat. Phys.* **15**, 1279–1283 (2019).

13. G. P. Zhang, T. F. George, Controlling vibrational excitations in $C_{60}$ by laser pulse durations. *Phys. Rev. Lett.* **93**, 147401 (2004).

14. M. Fischer, J. Handt, G. Seifert, R. Schmidt, Orientation dependence of energy absorption and relaxation dynamics of $C_{60}$ in fs-laser pulses. *Phys. Rev. A* **88**, 061403 (2013).

15. V. R. Bhardwaj, P. B. Corkum, D. M. Rayner, Internal laser-induced dipole force at work in $C_{60}$ molecule. *Phys. Rev. Lett.* **91**, 203004 (2003).

16. M. Boyle, T. Laarmann, I. Shchatsinin, C. P. Schulz, I. V. Hertel, Fragmentation dynamics of fullerenes in intense femtosecond-laser fields: Loss of small neutral fragments on a picosecond time scale. *J. Chem. Phys.* **122**, 181103 (2005).

17. T. Laarmann, I. Shchatsinin, A. Stalmashonak, M. Boyle, N. Zhavoronkov, J. Handt, R. Schmidt, C. P. Schulz, I. V. Hertel, Control of giant breathing motion in $C_{60}$ with temporally shaped laser pulses. *Phys. Rev. Lett.* **98**, 058302 (2007).

18. H. O. Jeschke, M. E. Garcia, J. Alonso, Nonthermal fragmentation of $C_{60}$. *Chem. Phys. Lett.* **352**, 154–162 (2002).

19. S. L. Dexheimer, D. M. Mittleman, R.W. Schoenlein, W. Vareka, X.-D. Xiang, A. Zettl, C.V. Shank, “Ultrafast dynamics of solid $C_{60}$,” in *Eighth International Conference on Ultrafast Phenomena*. Technical Digest Series (Optica Publishing Group, 1992).

20. K. Nakai, H. Kono, Y. Sato, N. Niitsu, R. Sahnoun, M. Tanaka, Y. Fujimura, Ab initio molecular dynamics and wavepacket dynamics of highly charged fullerene cations produced with intense near-infrared laser pulses. *Chem. Phys.* **338**, 127–134 (2007).

21. H. Fuest, Y. H. Lai, C. I. Blaga, K. Suzuki, J. Xu, P. Rupp, H. Li, P. Wnuk, P. Agostini, K. Yamazaki, M. Kanno, H. Kono, M. F. Kling, L. F. DiMauro, Diffractive imaging of $C_{60}$ structural deformations induced by intense femtosecond midinfrared laser fields. *Phys. Rev. Lett.* **122**, 053002 (2019).

22. T. Gorkhover, S. Schorb, R. Coffee, M. Adolph, L. Foucar, D. Rupp, A. Aquila, J. D. Bozek, S. W. Epp, B. Erk, L. Gumprecht, L. Holmegaard, A. Hartmann, R. Hartmann, G. Hauser, P. Holl, A. Hömke, P. Johnsson, N. Kimmel, K.-U. Kühnel, M. Messerschmidt, C. Reich, A. Rouzée, B. Rudek, C. Schmidt, J. Schulz, H. Soltau, S. Stern, G. Weidenspointner, B. White, J. Küpper, L. Strüder, I. Schlichting, J. Ullrich, D. Rolles, A. Rudenko, T. Möller, C. Bostedt, Femtosecond and nanometre visualization of structural dynamics in superheated nanoparticles. *Nat. Photon.* **10**, 93–97 (2016).

23. M. P. Minitti, J. M. Budarz, A. Kirrander, J. S. Robinson, D. Ratner, T. J. Lane, D. Zhu, J. M. Glownia, M. Kozina, H. T. Lemke, M. Sikorski, Y. Feng, S. Nelson, K. Saita, B. Stankus, T. Northey, J. B. Hastings, P. M. Weber, Imaging molecular motion: Femtosecond x-ray scattering of an electrocyclic chemical reaction. *Phys. Rev. Lett.* **114**, 255501 (2015).

24. J. M. Glownia, A. Natan, J. P. Cryan, R. Hartsock, M. Kozina, M. P. Minitti, S. Nelson, J. Robinson, T. Sato, T. van Driel, G. Welch, C. Weninger, D. Zhu, P. H. Bucksbaum, Self-referenced coherent diffraction x-ray movie of ångstrom- and femtosecond-scale atomic motion. *Phys. Rev. Lett.* **117**, 153003 (2016).

25. K. Haldrup, G. Levi, E. Biasin, P. Vester, M. G. Laursen, F. Beyer, K. S. Kjær, T. Brandt van Driel, T. Harlang, A. O. Dohn, R. J. Hartsock, S. Nelson, J. M. Glownia, H. T. Lemke, M. Christensen, K. J. Gaffney, N. E. Henriksen, K. B. Møller, M. M. Nielsen, Ultrafast x-ray scattering measurements of coherent structural dynamics on the ground-state potential energy surface of a diplatinum molecule. *Phys. Rev. Lett.* **122**, 063001 (2019).

26. B. Stankus, H. Yong, N. Zotev, J. M. Ruddock, D. Bellshaw, T. J. Lane, M. Liang, S. Boutet, S. Carbajo, J. S. Robinson, W. du, N. Goff, Y. Chang, J. E. Koglin, M. P. Minitti, A. Kirrander, P. M. Weber, Ultrafast x-ray scattering reveals vibrational coherence following rydberg excitation. *Nat. Chem.* **11**, 716–721 (2019).

27. A. Guinier, G. Fournet, *Small-Angle Scattering of X-rays* (John Wiley & Sons, Inc., New York, 1st ed., 1955).

28. R. Ehlich, M. Westerburg, E. E. B. Campbell, Fragmentation of fullerenes in collisions with atomic and molecular targets. *J. Chem. Phys.* **104**, 1900–1911 (1996).

29. C. Rose-Petruck, K. J. Schafer, K. R. Wilson, C. P. J. Barty, Ultrafast electron dynamics and inner-shell ionization in laser driven clusters. *Phys. Rev. A* **55**, 1182–1190 (1997).

30. P. B. Corkum, Plasma perspective on strong field multiphoton ionization. *Phys. Rev. Lett.* **71**, 1994–1997 (1993).

31. J. Kou, V. Zhakhovskii, S. Sakabe, K. Nishihara, S. Shimizu, S. Kawato, M. Hashida, K. Shimizu, S. Bulanov, Y. Izawa, Y. Kato, N. Nakashima, Anisotropic coulomb explosion of $C_{60}$ irradiated with a high-intensity femtosecond laser pulse. *J. Chem. Phys.* **112**, 5012–5020 (2000).

32. E. E. B. Campbell, R. D. Levine, Delayed ionization and fragmentation en route to thermionic emission: Statistics and dynamics. *Annu. Rev. Phys. Chem.* **51**, 65–98 (2000).

33. R. Boll, J. M. Schäfer, B. Richard, K. Fehre, G. Kastirke, Z. Jurek, M. S. Schöffler, M. M. Abdullah, N. Anders, T. M. Baumann, S. Eckart, B. Erk, A. de Fanis, R. Dörner, S. Grundmann, P. Grychtol, A. Hartung, M. Hofmann, M. Ilchen, L. Inhester, C. Janke, R. Jin, M. Kircher, K. Kubicek, M. Kunitski, X. Li, T. Mazza, S. Meister, N. Melzer, J. Montano, V. Music, G. Nalin, Y. Ovcharenko, C. Passow, A. Pier, N. Rennhack, J. Rist, D. E. Rivas, D. Rolles, I. Schlichting, L. P. H. Schmidt, P. Schmidt, J. Siebert, N. Strenger, D. Trabert, F. Trinter, I. Vela-Perez, R. Wagner, P. Walter, M. Weller, P. Ziolkowski, S. K. Son, A. Rudenko, M. Meyer, R. Santra, T. Jahnke, X-ray multiphoton-induced Coulomb explosion images complex single molecules. *Nat. Phys.* **18**, 423–428 (2022).

34. X. Li, R. Boll, P. Vindel-Zandbergen, J. González-Vázquez, D. E. Rivas, S. Bhattacharyya, K. Borne, K. Chen, A. de Fanis, B. Erk, R. Forbes, A. E. Green, M. Ilchen, B. Kaderiya, E. Kukk, H. V. S. Lam, T. Mazza, T. Mullins, B. Senfftleben, F. Trinter, S. Usenko, A. S. Venkatachalam, E. Wang, J. P. Cryan, M. Meyer, T. Jahnke, P. J. Ho, D. Rolles, A. Rudenko, Imaging a light-induced molecular elimination reaction with an X-ray free-electron laser. *Nat. Commun.* **16**, 7006 (2025).

35. B. Richard, R. Boll, S. Banerjee, J. M. Schäfer, Z. Jurek, G. Kastirke, K. Fehre, M. S. Schöffler, N. Anders, T. M. Baumann, S. Eckart, B. Erk, A. de Fanis, R. Dörner, S. Grundmann, P. Grychtol, M. Hofmann, M. Ilchen, M. Kircher, K. Kubicek, M. Kunitski, X. Li, T. Mazza, S. Meister, N. Melzer, J. Montano, V. Music, Y. Ovcharenko, C. Passow, A. Pier, N. Rennhack, J. Rist, D. E. Rivas, D. Rolles, I. Schlichting, L. P. H. Schmidt, P. Schmidt, D. Trabert, F. Trinter, R. Wagner, P. Walter, P. Ziolkowski, A. Rudenko, M. Meyer, R. Santra, L. Inhester, T. Jahnke, Imaging collective quantum fluctuations of the structure of a complex molecule. *Science* **389**, 650–654 (2025).

36. Y. Inubushi, G. Yamaguchi, J. Yamada, Y. Kubota, I. Inoue, T. Osaka, T. Yabuuchi, K. Tono, M. Yabashi, Development of portable nanofocusing optics for X-ray free-electron laser pulses. *J. Synchrotron Rad.* **32**, 534–538 (2025).

37. P. Emma, R. Akre, J. Arthur, R. Bionta, C. Bostedt, J. Bozek, A. Brachmann, P. Bucksbaum, R. Coffee, F. J. Decker, Y. Ding, D. Dowell, S. Edstrom, A. Fisher, J. Frisch, S. Gilevich, J. Hastings, G. Hays, P. Hering, Z. Huang, R. Iverson, H. Loos, M. Messerschmidt, A. Miahnahri, S. Moeller, H. D. Nuhn, G. Pile, D. Ratner, J. Rzepiela, D. Schultz, T. Smith, P. Stefan, H. Tompkins, J. Turner, J. Welch, W. White, J. Wu, G. Yocky, J. Galayda, First lasing and operation of an ångstrom-wavelength free-electron laser. *Nat. Photon.* **4**, 641–647 (2010).

38. T. Osipov, C. Bostedt, J. C. Castagna, K. R. Ferguson, M. Bucher, S. C. Montero, M. L. Swiggers, R. Obaid, D. Rolles, A. Rudenko, J. D. Bozek, N. Berrah, The lamp instrument at the linac coherent light source free-electron laser. *Rev. Sci. Instrum.* **89**, 035112 (2018).

39. J. Tersoff, Modeling solid-state chemistry: Interatomic potentials for multicomponent systems. *Phys. Rev. B* **39**, 5566–5568 (1989).

40. K. R. Ferguson, M. Bucher, J. D. Bozek, S. Carron, J. C. Castagna, R. Coffee, G. I. Curiel, M. Holmes, J. Krzywinski, M. Messerschmidt, M. Minitti, A. Mitra, S. Moeller, P. Noonan, T. Osipov, S. Schorb, M. Swiggers, A. Wallace, J. Yin, C. Bostedt, The atomic, molecular and optical science instrument at the linac coherent light source. *J. Synchrotron Radiat.* **22**, 492–497 (2015).

41. L. Strüder, S. Epp, D. Rolles, R. Hartmann, P. Holl, G. Lutz, H. Soltau, R. Eckart, C. Reich, K. Heinzinger, C. Thamm, A. Rudenko, F. Krasniqi, K. U. Kühnel, C. Bauer, C. D. Schröter, R. Moshammer, S. Techert, D. Miessner, M. Porro, O. Hälker, N. Meidinger, N. Kimmel, R. Andritschke, F. Schopper, G. Weidenspointner, A. Ziegler, D. Pietschner, S. Herrmann, U. Pietsch, A. Walenta, W. Leitenberger, C. Bostedt, T. Möller, D. Rupp, M. Adolph, H. Graafsma, H. Hirsemann, K. Gärtner, R. Richter, L. Foucar, R. L. Shoeman, I. Schlichting, J. Ullrich, Large-format, high-speed, x-ray pnCCDs combined with electron and ion imaging spectrometers in a multipurpose chamber for experiments at 4th generation light sources. *Nucl. Instrum. Methods Phys. Res. A* **614**, 483–496 (2010).

42. M. P. Minitti, J. S. Robinson, R. N. Coffee, S. Edstrom, S. Gilevich, J. M. Glownia, E. Granados, P. Hering, M. C. Hoffmann, A. Miahnahri, D. Milathianaki, W. Polzin, D. Ratner, F. Tavella, S. Vetter, M. Welch, W. E. White, A. R. Fry, Optical laser systems at the Linac Coherent Light Source. *J. Synchrotron Radiat.* **22**, 526–531 (2015).

43. X. Lu, L. Feng, T. Akasaka, S. Nagase, Current status and future developments of endohedral metallofullerenes. *Chem. Soc. Rev*. **41**, 7723–7760 (2012).

44. K. R. Ferguson, M. Bucher, T. Gorkhover, S. Boutet, H. Fukuzawa, J. E. Koglin, Y. Kumagai, A. Lutman, A. Marinelli, M. Messerschmidt, K. Nagaya, J. Turner, K. Ueda, G. J. Williams, P. H. Bucksbaum, C. Bostedt, Transient lattice contraction in the solid-to-plasma transition. *Sci. Adv*. **2**, e1500837 (2016).

45. K. R. Ferguson, Crystal structure determination of xenon nanoparticles and x-ray induced transient lattice contraction in the solid-to-plasma transition, Ph.D. thesis, Stanford University (2016). https://stacks.stanford.edu/file/druid:qk328xg1417/Submitted-Dissertation-augmented.pdf.

46. M. Harmand, R. Coffee, M. R. Bionta, M. Chollet, D. French, D. Zhu, D. M. Fritz, H. T. Lemke, N. Medvedev, B. Ziaja, S. Toleikis, M. Cammarata, Achieving few-femtosecond time-sorting at hard x-ray free-electron lasers. *Nat. Photon.* **7**, 215–218 (2013).

47. S. Schorb, T. Gorkhover, J. P. Cryan, J. M. Glownia, M. R. Bionta, R. N. Coffee, B. Erk, R. Boll, C. Schmidt, D. Rolles, A. Rudenko, A. Rouzee, M. Swiggers, S. Carron, J. C. Castagna, J. D. Bozek, M. Messerschmidt, W. F. Schlotter, C. Bostedt, X-ray–optical cross-correlator for gas-phase experiments at the Linac Coherent Light Source free-electron laser. *Appl. Phys. Lett.* **100**, 121107 (2012).

48. B. F. Murphy, T. Osipov, Z. Jurek, L. Fang, S. K. Son, M. Mucke, J. H. D. Eland, V. Zhaunerchyk, R. Feifel, L. Avaldi, P. Bolognesi, C. Bostedt, J. D. Bozek, J. Grilj, M. Guehr, L. J. Frasinski, J. Glownia, D. T. Ha, K. Hoffmann, E. Kukk, B. K. McFarland, C. Miron, E. Sistrunk, R. J. Squibb, K. Ueda, R. Santra, N. Berrah, Femtosecond x-ray-induced explosion of $C_{60}$ at extreme intensity. *Nat. Comm.* **5**, 4281 (2014).

49. U. Saalmann, C. Siedschlag, J. M. Rost, Topical review: Mechanisms of cluster ionization in strong laser pulses. *J. Phys. B At. Mol. Opt. Phys.* **39**, R39 (2006).

50. S. G. Kim, D. Tománek, Melting the fullerenes: A molecular dynamics study. *Phys. Rev. Lett.* **72**, 2418–2421 (1994).

51. A. D. Bandrauk, S. Chelkowski, N. H. Shon, Measuring the electric field of few-cycle laser pulses by attosecond cross correlation. *Phys. Rev. Lett.* **89**, 283903 (2002).

52. H. Li, B. Mignolet, G. Wachter, S. Skruszewicz, S. Zherebtsov, F. Süßmann, A. Kessel, S. A. Trushin, N. G. Kling, M. Kübel, B. Ahn, D. Kim, I. Ben-Itzhak, C. L. Cocke, T. Fennel, J. Tiggesbäumker, K. H. Meiwes-Broer, C. Lemell, J. Burgdörfer, R. D. Levine, F. Remacle, M. F. Kling, Coherent electronic wave packet motion in $C_{60}$ controlled by the waveform and polarization of few-cycle laser fields. *Phys. Rev. Lett.* **114**, 123004 (2015).

53. B. Mignolet, R. D. Levine, F. Remacle, Control of electronic dynamics visualized by angularly resolved photoelectron spectra: A dynamical simulation with an IR pump and xuv attosecond-pulse-train probe. *Phys. Rev. A* **89**, 021403 (2014).

54. T. Yanai, Y. Kurashige, D. Ghosh, G. K.-L. Chan, Accelerating convergence in iterative solution for large-scale complete active space self-consistent-field calculations. *Int. J. Quantum Chem.* **109**, 2178–2190 (2009).

55. B. Mignolet, J. O. Johansson, E. E. B. Campbell, F. Remacle, Probing rapidly-ionizing super-atom molecular orbitals in $C_{60}$: A computational and femtosecond photoelectron spectroscopy study. *ChemPhysChem* **14**, 3332–3340 (2013).

56. B. Mignolet, R. D. Levine, F. Remacle, Localized electron dynamics in attosecond-pulse-excited molecular systems: Probing the time-dependent electron density by sudden photoionization. *Phys. Rev. A* **86**, 053429 (2012).

57. G. M. Seabra, I. G. Kaplan, V. G. Zakrzewski, J. V. Ortiz, Electron propagator theory calculations of molecular photoionization cross sections: The first-row hydrides. *J. Chem. Phys.* **121**, 4143–4155 (2004).
